# CHD1L augments autophagy-mediated migration of hepatocellular carcinoma through targeting *ZKSCAN3*

**DOI:** 10.1038/s41419-021-04254-x

**Published:** 2021-10-15

**Authors:** Xiaofeng Zhang, Yinshan Bai, Li Huang, Shanshan Liu, Yanxuan Mo, Wei Cheng, Guangliang Wang, Zhiming Cao, Xiaogang Chen, Huiqing Cui, Ling Qi, Lei Ma, Ming Liu, Xin-Yuan Guan, Ning-Fang Ma

**Affiliations:** 1 Affiliated Cancer Hospital and Institute of Guangzhou Medical University, Guangzhou, Guangdong China; 2 Guangzhou Municipal and Guangdong ProvincialKey Laboratory of Protein Modification and Degradation, School of Basic Medical Sciences, Guangzhou Medical University, Guangzhou, Guangdong China; 3 School of Life Science and Engineering, Foshan University, Foshan, Guangdong China; 4 The Sixth Affiliated Hospital of Guangzhou Medical University, Qingyuan People’s Hospital, Qingyuan, Guangdong China; 5 State Key Laboratory of Oncology in South China, Collaborative Innovation Center for Cancer Medicine, Cancer Center, Sun Yat-sen University, Guangzhou, Guangdong China; 6 Department of Clinical Oncology, Center for Cancer Research, and State Key Laboratory for Liver Research, University of Hong Kong, Pok Fu Lam, Hong Kong; 7 Department of Histology and Embryology, School of Basic Medical Sciences, Guangzhou Medical University, Guangzhou, Guangdong China

**Keywords:** Oncogenes, Focal adhesion

## Abstract

Autophagy is an important biological process in normal cells. However, how it affects tumor progression still remains poorly understood. Herein, we demonstrated that the oncogenic protein Chromodomain-helicase-DNA-binding-protein 1-like gene (CHD1L) might promote HCC cells migration and metastasis through autophagy. CHD1L could bind to the promotor region of Zinc finger with KRAB and SCAN domain 3 (*ZKSCAN3)*, a pivotal autophagy suppressor, and inhibit its transcription. We established inducible *CHD1L* conditional knockout cell line (CHD1L*-*iKO cell) and found that the deletion of CHD1L significantly increased ZKSCAN3 expression both at mRNA and protein level. Deletion of CHD1L impaired the autophagic flux and migration of HCC cells, while specifically inhibiting *ZKSCAN3* blocked these effects. Further exploration demonstrated that the enhanced tumor cell migration and metastasis induced by CHD1L was mediated through ZKSCAN3*-*induced autophagic degradation of Paxillin. In summary, we have characterized a previously unknown function of CHD1L in regulating tumor migration via ZKSCAN3-mediated autophagy in HCC. Further inhibition of CHD1L and its downstream autophagy signaling might shed new light on cancer therapeutics.

## Introduction

Autophagy (Macro autophagy) is generally considered as a cell survival mechanism that supplies nutrients and removes the aggregated proteins or damaged organelles of cells to ensure the turnover of obsolete cellular components [1]. Growing evidences showed a powerful potential of autophagy in promoting tumorigenesis and metastasis [2–6]. Further elucidation of the molecular mechanisms of autophagy activity during tumor malignant progression will help understand tumor pathogenesis and provide novel strategies for precision medication.

Chromodomain-helicase-DNA-binding-protein 1-like gene (*CHD1L)* was identified to be frequently amplified at chromosome 1q21 in many cancers and characterized as a critical oncogenic driver in our previous studies [7–9]. Growing evidences demonstrated that CHD1L was highly expressed in most types of malignancy including hepatocellular carcinoma [10–13], lung cancer [14], breast cancer [15], gastric cancer [16], colorectal cancer [17], bladder cancer [18], and ovarian cancer [19]. A very recent study suggested CHD1L as an important regulator of PARP inhibitor potency [20, 21]. In addition, first-in-class CHD1L small molecular inhibitors are already under intensive investigation in pre-clinical models [22]. All these findings proposed CHD1L as a very promising drug target for cancer treatment. Our recent evidences from chromatin Immunoprecipitation (ChIP) of CHD1L followed by high-throughput sequencing indicated that CHD1L might also be closely associated with tumor cell autophagy. A critical autophagy regulator, Zinc finger with KRAB and SCAN domain 3 (*ZKSCAN3*), was identified to be a potential CHD1L downstream target with high rank [23]. ZKSCAN3 belongs to the family of zinc finger transcription factors, and contains the CHD1L-binding motif upstream to its transcription start site (TSS). Overexpression of *ZKSCAN3* was reported to negatively regulate the genes correlated with autophagosome formation and lysosome production [24–27]. In the present study, we demonstrated that CHD1L was closely involved in autophagy regulation. CHD1L negatively controlled the transcription of *ZKSCAN3* and prevented the inhibitory role of *ZKSCAN3* in tumor cell autophagy. Deletion of CHD1L blocked autophagosome formation as well as autophagic flux. Overexpression of CHD1L promoted HCC cells migration potentially through facilitating the autophagic degradation of focal adhesion (FA) protein Paxillin. We proved that the CHD1L/ZKSCAN3 axis was important in cell autophagy-induced HCC tumor migration. Further targeting the critical regulators in HCC cells autophagy might be a novel promising strategy in cancer treatment.

## Results

### Blocking autophagy in HCC cells impairs the role of CHD1L in tumor cell migration and metastasis

**Fig. 1:**
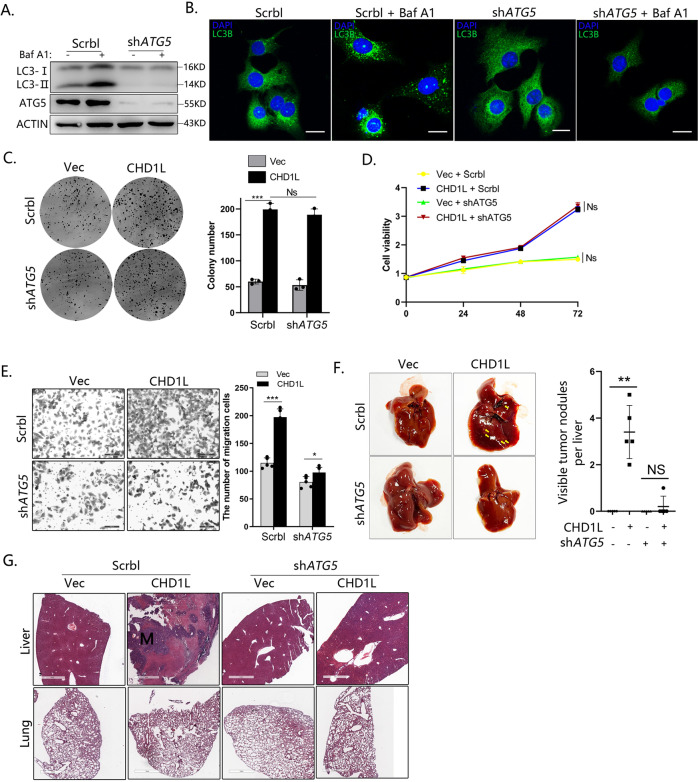
Blocking autophagy in HCC cells impair the role of CHD1L to cell metastasis but not cell growth. **A** Western blot for ATG5 in Scrbl shRNA and *ATG5* shRNA-expressing QGY-7703 clones in the presence or absence of 80 nM bafA1 (6 h). **B** Immunofluorescence for endogenous LC3B. **C** Crystal violet staining assays of Scrbl shRNA and ATG5 shRNA-expressing QGY-7703 clones in the indicated group for 2 weeks. **D** Cell growth in complete medium over 72 h. Mean ± SD, *n* = 3. **E** Migration assay and related quantification for Scrbl shRNA and ATG5 shRNA-expressing QGY-7703 clones in Vector or forced CHD1L group. (***p < 0.001, Ns no significant; Student’s *t* test; Mean ± SD, *n* = 5/group). **F** Representative overall picture of livers of mice with Scrbl shRNA and ATG5- shRNA in Vector or forced CHD1L tumors 6 weeks post-spleen injection. Mean number of livers metastases in mice with control or autophagy-deficient in Vector or forced CHD1L tumors at 6 weeks (**p < 0.01, Ns no significant; Student’s *t* test; Mean ± SD, *n* = 5/group). **G** Representative H&E-stained sections of lungs and livers of mice with Scrbl shRNA and ATG5- shRNA in Vector or forced CHD1L tumors 6-w post-spleen injection.

Considering that CHD1L could induce multiple malignant phenotypes of tumor cells including cell growth and metastasis, how cell autophagy was involved in malignant transformation was first investigated. To block cell autophagy, short hairpin (sh)-RNA (shRNA)-mediated knockdown of *ATG5*, autophagy-regulatory proteins required for autophagosome formation, was performed in HCC cells in accordance with published guidelines [28]. Bafilomycin A1 (Baf A1), a late-stage autophagy inhibitor that prevents the fusion of autophagosome to autolysosome, was used to block the autophagic flux. As shown in Fig. 1A, the autophagosome marker LC3B-II was significantly increased after Baf A1 treatment. However, this was abrogated when ATG5 was silenced. Immunofluorescence (IF) for endogenous LC3B revealed that the cell punctae was increased in the presence of Baf A1. However, in autophagy-deficient cells with ATG5 knocked down, only diffused cytoplasmic staining of LC3B was observed with or without Baf A1 treatment (Fig. 1B). These findings indicated that ATG5 is required for HCC tumor cell autophagy. Next, we explored whether the lack of autophagy will indeed affect the tumorigenicity of CHD1L on HCC. We stably transfected CHD1L or Vector control in both Scrbl and shATG5 clones in QGY-7703 cells, respectively (Fig. S1A). Although marked inhibition of autophagy was found in ATG5-deficient QGY-7703 cells, the effects of autophagy on either cell growth or viability induced by CHD1L were minor (Fig. 1C, D). However, the lack of autophagy significantly impaired the migratory ability of HCC cells induced by overexpression of CHD1L (Fig. 1E). Similar results were obtained upon addition of autophagosome-lysosome fusion inhibitors Baf A1 and Chloroquine (Fig. S1B,C). Furthermore, we additionally used 3-MA, a common early inhibitor of autophagy, and the similar results were obtained (Fig. S1D, E). To further corroborate the potential role of autophagy in HCC tumor metastasis, QGY-7703 cells stably transfected with Scrbl or shATG5 were injected into mice spleen to establish a tumor metastasis model as previously described [29]. Overexpression of CHD1L dramatically induced liver metastasis of QGY-7703 cells in vivo, but this was significantly abolished in autophagy-deficient cells (Fig. 1F, G). Taken together, these results demonstrated that autophagy is required for HCC tumor cell migration and metastasis induced by CHD1L.

### CHD1L promotes autophagy in HCC

**Fig. 2:**
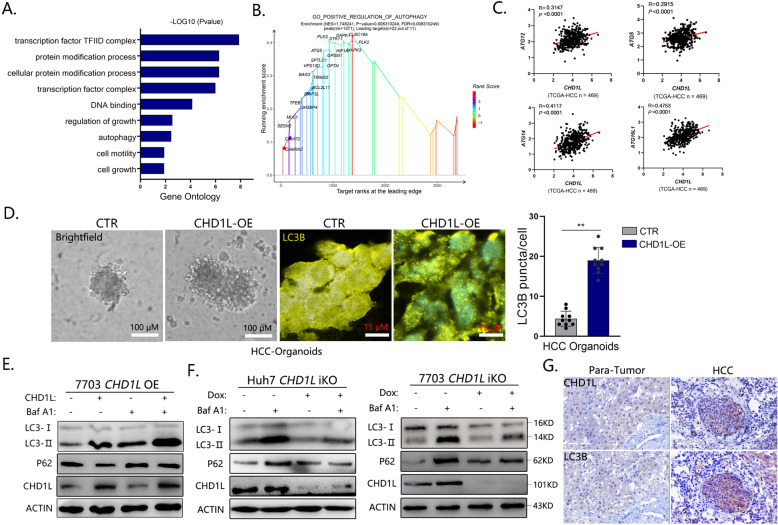
CHD1L promotes autophagy in HCC. **A** Enrichment of the gene ontology (GO) terms of differential mRNAs in CHD1L- vs vector-QGY-7703 clones. **B** Performance of GSEA based on the differential genes in CHD1L- vs vector-QGY-7703 clones. Enrichment analysis was performed on the indicated gene sets. **C** Correlation plots show expression of indicated genes and CHD1L in 469 primary HCC tumor and para-tumor samples analyzed using RNA-sequencing in TCGA. **D** Bright light and immunofluorescence for endogenous LC3B images of patient-derived organoid (PDOs) established from HCC. Cystic PDO structures were recognized starting on day 6. Bar was as indicated, quantification of LC3B puncta per cell for Vector or forced CHD1L group. (**p < 0.01; Student’s *t* test; Mean ± SD, *n* = 10/group). **E** WB analysis for changes in LC3 conversion and P62 level affected by overexpression of CHD1L in the presence or absence of 80 nM Baf A1 (6 h). The QGY-7703 cells were transiently transfected with GFP-tagged CHD1L expressing constructs or the control vector. LC3-I, non-lipidated LC3; LC3-II, lipidated LC3. The β-actin protein was as a loading control. **F** WB analysis for changes in LC3 conversion and P62 level affected by CHD1L-deficient in the presence or absence of 80 nM bafA1 (6 h) in Huh7 and QGY-7703-iKO cells. **G** Representative IHC images for the expression of CHD1L and LC3B in primary HCC tumor.

Our previous data from high-throughput sequencing of CHD1L overexpressing cell indicated that CHD1L is closely associated with autophagy. Gene ontology (GO) and Gene Set Enrichment Analysis (GSEA) further supported that the enrichment patterns of CHD1L-overexpressed differential genes (DEGs) signatures were related to POSITIVE REGULATION OF AUTOPHAGY (Fig. 2A, B). In addition, the expression of several autophagy critical components such as ATG12, ATG14, et al. were positively correlated with the expression of CHD1L in primary HCC samples from the TCGA database (Fig. 2C). To further explore the effects of CHD1L on HCC tumor cell autophagy, primary HCC tissues were cultured in a 3D organoid system according to published protocols [30] (Fig. 2D Brightfield channel). Forced expression of CHD1L was induced by lentivirus-mediated transfection. IF for endogenous LC3B (autophagy specific marker) revealed numerous punctae in CHD1L overexpression cells compared with the diffused cytoplasmic staining or few of granular staining in control cells. To further investigate the role of CHD1L on autophagy, western blot was performed in QGY-7703 cells stably transfected with *CHD1L* or Vector control. During autophagy progression, cytosolic LC3-I will conjugate to phosphatidylethanolamine to form LC3-II which finally incorporate into the autophagosomal membrane [3–5]. As shown in Fig. 2E, overexpression of *CHD1L* significantly increased LC3-II when compared with the vector control, and this effect was augmented in the presence of Baf A1 treatment. P62 serves as a scaffold by binding with ubiquitinated cellular cargoes and autophagosomal membrane protein LC3, facilitating substrate degradation of autophagy. Protein aggregates formed by p62 are often used as reporters of retarded autophagy activity. The decrease in P62 while the increase in LC3-II is considered increase of autophagic flux. In addition, accumulation of p62 could be a good indicator of autophagy suppression [31–33]. Meanwhile, we found p62 was decreased upon CHD1L expression without Baf A1 treatment. These results indicated that CHDL1 might positively regulate tumor cell autophagy in HCC. Dox inducible *CHD1L* conditional knockout liver cancer cell lines were further established in Huh7 and QGY-7703 cells (Huh7-iKO and 7703-iKO) In contrast, although the cells without Dox induce exhibited visible autophagic flux with Baf A1 treatment (compare lane 1 and 2, Fig. 2F), as measured by increased autophagosome marker LC3-II, cells (Huh7-iKO and 7703-iKO cells) with CHD1L deletion failed to do so in the presence of Baf A1 (Compare lane 3 and 4, Fig. 2F). Meanwhile, we found p62 was increased upon CHD1L deletion without Baf A1 treatment (compare lane 1 and 3) (Fig. 2F). In addition, immunochemistry (IHC) of both LC3B and CHD1L staining were increased in HCC tumor samples compared with the corresponding para-tumor liver tissues (Fig. 2G). All these findings indicated that CHD1L is required for tumor cell autophagy.

### Knockout of *CHD1L* significantly blocked the autophagic flux induced by starvation in HCC cells

**Fig. 3:**
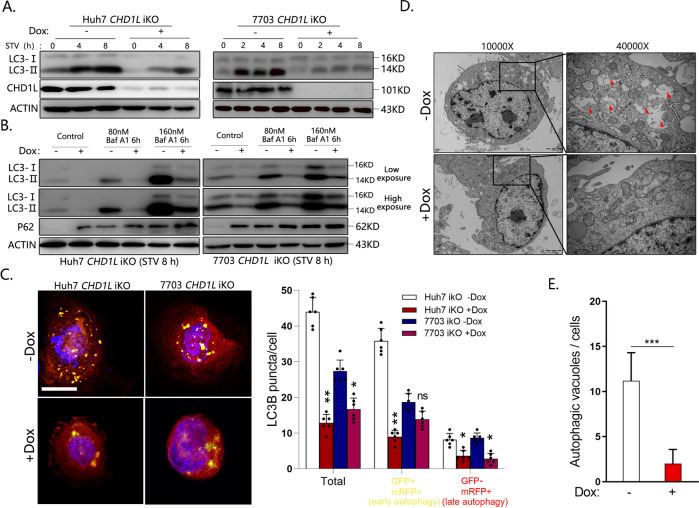
Knockout of CHD1L significantly blocked the autophagic flux induced by starvation in HCC cells. **A** WB analysis for starvation-induced changes in LC3 conversion in Dox (−/+)-induced Huh7 and QGY-7703-iKO cells. **B** WB analysis of changes induced by CHD1L ablation in LC3 conversion in Huh7 and QGY-7703-iKO cells treated with Baf A1 at indicated concentration. **C** Representative images showing starvation-induced mRFP-LC3 and GFP-mRFP-LC3 puncta accumulation in response to CHD1L deletion in Huh7 and QGY-7703-iKO cells. Quantification of mRFP-LC3 and GFP-mRFP-LC3 puncta per cell. Bar = 10 μM (*p < 0.05; **p < 0.01, ns No significant; Student’s *t* test; Data indicate mean ± SD; *n* = 5). **D** Ultrastructural evidence of autophagy induced by starvation in the indicated cells with CHD1L expression or ablation. The AVd indicate late/degradative autophagolysosomes. Magnification ×10,000–40,000, scale bar represents as shown. **E** Quantification for autophagic vacuoles as described in “Materials and methods”. (***p < 0.001, Student’s *t* test; Data indicate mean ± SD; *n* = 10). Images/immunoblots are representative of three independent experiments.

The basic autophagy of tumor cells will be greatly enhanced to maintain cell survival under stressed conditions, such as hypoxia or nutritional deprivation. Nutrient limitation often occurs during tumor development [34]. We therefore assessed whether CHD1L was important for the starvation-induced autophagy response. As shown in results, serum starvation induced a direct increase in LC3-II level, but was significantly blocked by *CHD1L* ablation (Fig. 3A). Depletion of CHD1L also significantly blocked the autophagic flux induced by Baf A1 treatment under stress conditions (Fig. 3B). To further corroborate these findings, a vector encoding LC3 fused to monomeric red-fluorescence protein and GFP in tandem (GFP-mRFP-LC3) was constructed according to the previous study [35]. In autophagosomes, both red and green fluorescence signals will appear. In contrast, green fluorescence is diminished due to the labile of GFP protein under acidic conditions in the autophagolysosomes, and only the monomeric red fluorescent protein is stable, which results in yielding single red fluorescence. Huh7-iKO and 7703-iKO cells were transfected with GFP-mRFP-LC3 vector. After deletion of CHD1L, both signals representing early autophagy (yellow) and late autophagy (red) were significantly decreased, suggesting CHD1L is important in autophagic flux in HCC (Fig. 3C). Finally, transmission electron microscopy (TEM) assay was performed to observe the autophagic vacuole, the golden standard for identification of autophagy. As shown in Fig. 3D, E, abundant late/degradative autophagic vacuole (AVd) appeared in 7703-iKO cells without Dox treatment, but decreased significantly upon Dox induction, which further confirmed the role of CHD1L in autophagy.

### CHD1L negatively regulates ZKSCAN3 transcription

**Fig. 4:**
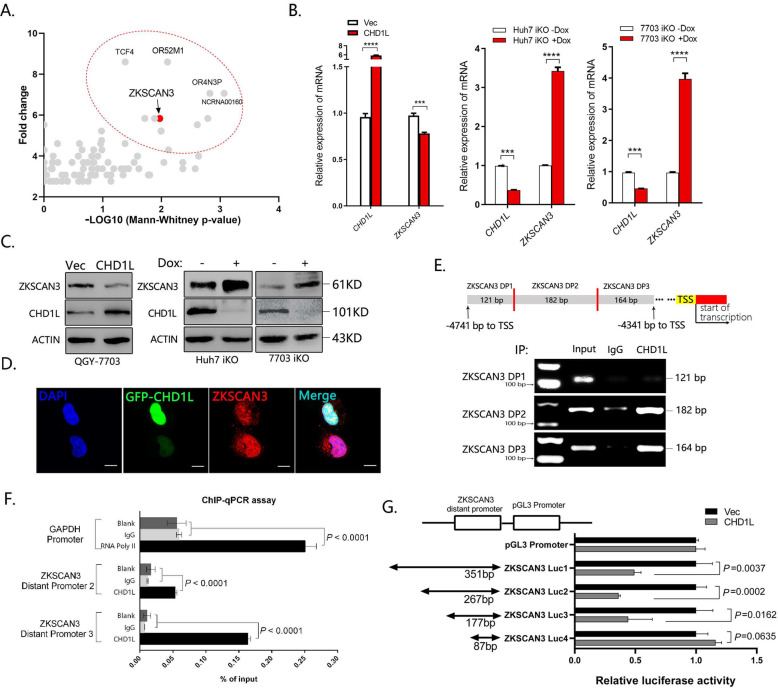
CHD1L inhibits ZKSCAN3 transcription. **A** Visualization results of CHIP-seq by anti-CHD1L. Quantitative real-time-PCR (qRT–PCR) and WB were performed to determine the impact of CHD1L knockout or overexpression on mRNA (**B**) and protein (**C**) expression of ZKSCAN3 in Huh7 and QGY-7703 cells (***p < 0.001, ****p < 0.0001, Student’s *t* test). The Dox-induced CRISPR/Cas9 system (1 μg/ml, 72 h) was applied to knockout *CHD1L* in Huh7 and QGY-7703 cells, and the β-actin was used as loading control (referred to as ACTIN). **D** Representative images of CHD1L and ZKSCAN3 immunofluorescence staining in QGY-7703 cells transfected with GFP-tagged CHD1L. The stained cells were observed using confocal fluorescence microscopy. Scale bar represents: 10 μM. Images/immunoblots are representative of three independent experiments. Dox, Doxycycline. **E**, **F** Predicted CHD1L-binding site (ZKSCAN3 DP1, DP2 and DP3) within a region between 4741 to 4341 bp upstream of the TSS of *ZKSCAN3* was evaluated by ChIP assay with anti-CHD1L IP in QGY-7703 cells. Anti-IgG IP was used as a negative control (**E**). ChIP-PCR was used to validate the binding of CHD1L to the identified promoter region of *ZKSCAN3* in QGY-7703 cells (**F**). **G** Luciferase reporter assay in QGY-7703 cells using different fragments of ZKSCAN3 promoter after transfection with CHD1L or Vector, *n* = 3 biologically independent samples; data shown are mean ± SD. A two-sided Student’s *t* test was used to generate *p* values.

We previously described CHD1L belongs to the SNF2-like family, containing a conserved SNF2_N domain, a helicase superfamily domain (HELICc), and a Macro domain, which is actively involved in regulating gene transcription [11–13]. ZKSCAN3, a recently identified transcriptional repressor of autophagy, was identified to be a potential CHD1L-binding target with high priority from the ChIP-sequencing data (Fig. 4A). As shown in Fig. 4B, C, deletion of *CHD1L* significantly upregulated ZKSCAN3 expression both at mRNA and protein level. In contrast, overexpression of *CHD1L* suppressed ZKSCAN3 expression both at mRNA and protein level. The expression of ZKSCAN3 was further detected by IF staining, and the results showed that weak or negative staining of ZKSCAN3 (red) was found in the nucleus of cells with strong CHD1L signaling (green). Conversely, high expression of ZKSCAN3 was found in CHD1L negative cells (Fig. 4D). These findings indicated that the expression of ZKSCAN3 was negatively correlated with CHD1L. The CHD1L-binding region displayed by CHIP-seq is from 4741 to 4341 bp upstream to the TSS of *ZKSCAN3* (Fig. 4E). To identify the peak binding region of CHD1L on ZKSCAN3, three pairs of primers covering different sites of the predicted regions were designed for ChIP-PCR assay. In accordance with the ChIP-sequencing data, the peak binding site of CHD1L was confirmed be located at the 4692–4341 bp region upstream to TSS of ZKSCAN3 (Fig. 4E, F). Putative CHD1L-binding fragments (fragment 1 (−4741 to −4341 bp), fragment 2 (−4608 to −4341 bp), fragment 3 (−4518 to −4341 bp), and fragment 4 (-4428 to -4341bp)) were constructed in pGL3 vectors, and transiently transfected into QGY-7703 cells in indicated combinations. The luciferase reporter assay indicated that the chromosomal region from −4741 to −4341 bp upstream to the TSS of ZKSCAN3 was involved in transcriptional suppression by CHD1L (Fig. 4G). All these results strongly indicated that CHD1L might negatively regulate ZKSCAN3 transcription.

### ZKSCAN3 is required for CHD1L-induced tumor cell autophagy

**Fig. 5:**
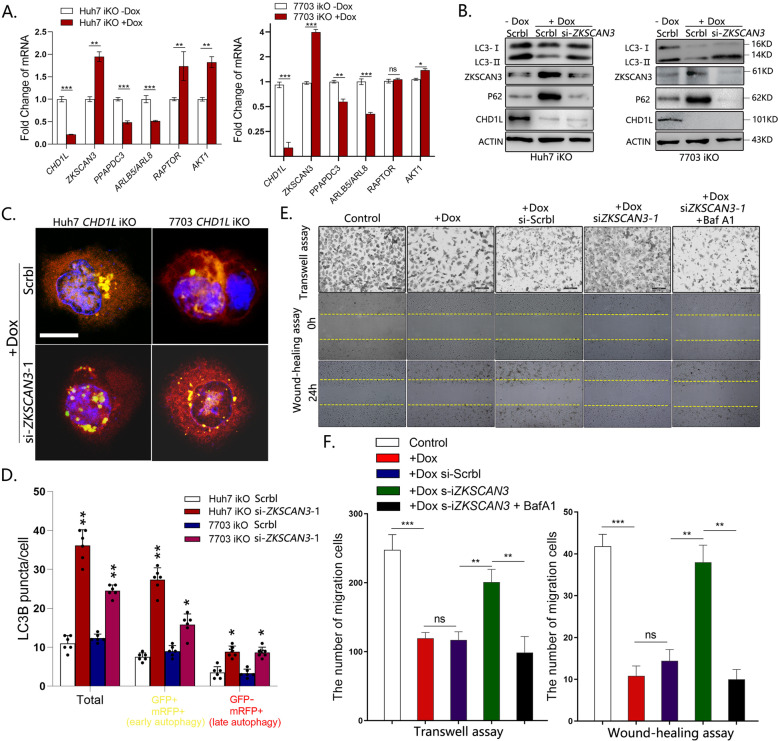
Silencing ZKSCAN3 rescue the lacked autophagy and migration due to deleting CHD1L in vitro. **A** Huh7 and QGY-7703-iKO cells were detected for expression of indicated ZKSCAN3 downstream autophagy-related genes by fluorescent quantitative RT-PCR (**P < 0.01; ***P < 0.001; Student’s *t* test). Values are shown as mean ± SD calculated from three parallel experiments. **B**. WB analysis for LC3 conversion changes in CHD1L deleted cells in response to ZKSCAN3 knockdown by si*ZKSCAN3*-1, compared to Scrbl control. **C**, **D** Representative images showing starvation-induced GFP-mRFP-LC3 and mRFP-LC3 puncta accumulation in CHD1L deleted cells in response to ZKSCAN3 knockdown. GFP-mRFP-LC3 and mRFP-LC3 puncta per cell were quantified. Scale bar represents: 10 μM (*p < 0.01; **p < 0.01; Student’s *t* test; Data indicate mean ± SD; *n* = 5). Images/immunoblots are representative of three independent experiments. **E, F.** QGY-7703 cells treated as indicated (with PBS control, Baf A1 (40 nM)) were plated in the upper chamber of the filters for 24 h and then, the cells migrated to the underside of the Transwell insert were counted, scale bar: 100 μm. Baf A1: 40 nM. QGY-7703 cells treated as indicated were subjected to wound-healing assay (Upper). Quantification of migration in Transwell assay and migrated area in wound-healing assay (Lower) (**p < 0.01; ***p < 0.001; ns no significant; Student *t* test; *n* = 5).

To explore if CHD1L acts through ZKSCAN3 to promote autophagy, siRNAs were used to specifically inhibit *ZKSCAN3* expression (Fig. S2A). Silencing of ZKSCAN3 significantly increased LC3-II and decreased P62 level in a dose-dependent manner (Fig. S2B). To test whether ZKSCAN3-regulated autophagy genes were also controlled by CHD1L, ZKSCAN3 downstream autophagy-related genes, including *PPAPDC3*, *ARLB5/ARL8* (positive regulator of autophagy) and *RAPTOR, AKT1* (negative regulator of autophagy) [23] were examined by qPCR in Huh7-iKO and 7703-iKO cells treated with or without Dox. As shown in the results, the downstream targets of ZKSCAN3 were also regulated by CHD1L in the same trend (Fig. 5A). We also examined the effect of CHD1L on the expression of mTOR signaling pathway, a conversed regulator in autophagy, No significant change was found, suggesting that mTOR signaling pathway may not be involved in CHD1L-regulated autophagy (Fig. S2C). To test whether autophagy attenuation induced by CHD1L deletion would be rescued by *ZKSCAN3* knockdown, *ZKSCAN3* specific siRNA was transfected into Huh7-iKO and 7703-iKO cells, respectively. As expected, deletion of CHD1L caused a significant decrease in LC3-II, and this could be rescued by *ZKSCAN3* knockdown (Fig. 5B). Similar results were also obtained in relevant cells upon Baf A1 treatment (Fig. S2D) In order to confirm whether the role of CHD1L in autophagic flux is dependent on ZKSCAN3, GFP-mRFP-LC3 construct was transfected into Huh7-iKO and 7703-iKO cells. As expected, knockdown of *ZKSCAN3* induced a higher ratio of yellow autophagosomes puncta and red autolysosomes puncta (Fig. 5C, D), indicating that the inhibited autophagic flux by depletion of *CHD1L* was reversed after downregulation of *ZKSCAN3*. To determine whether ZKSCAN3 is essential in CHD1L-induced HCC cell migration, the effects of ZKSCAN3 downregulation on HCC cell migration after CHD1L deletion were examined in transwell assay and wound-healing assay. As expected, (Fig. 5E, F), knockdown of ZKSCAN3 in cells lacking *CHD1L* alleviated its inhibitory role on cell migration, and this effect was abolished by blocking autophagy with Baf A1 treatment.

### Both enhanced autophagy level and migration ability on HCC cells mediated by CHD1L were diminished through overexpression of ZKSCAN3

**Fig. 6:**
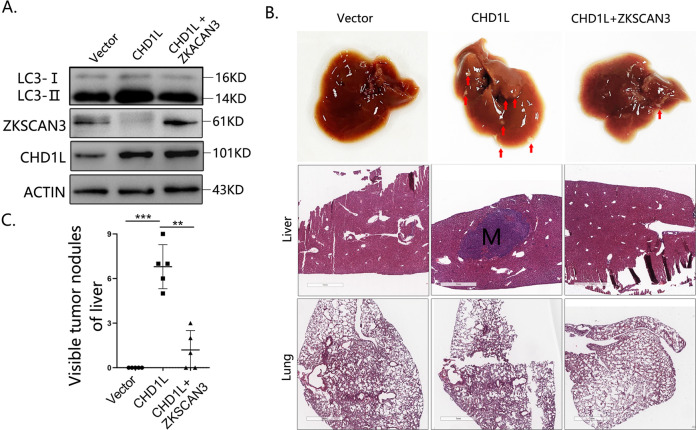
Both autophagy level and migration ability mediated by CHD1L were diminished through overexpression of ZKSCAN3 on HCC cells in vivo. **A** WB analysis for the changes of LC3 conversion, CHD1L, and ZKSCAN3 in QGY-7703 cells treated as indicated. **B** QGY-7703 cells stably transfected with CHD1L or CHD1L + ZKSCAN3 and their control lentivirus were intrasplenically inoculated into BALB/c nude mice for 6 weeks (The red arrow represents the visible metastasis). Representative histopathological examination images of the BALB/c nude mice liver and lung injected with the constructed QGY-7703-Vec, QGY-7703-CHD1L, and QGY-7703-CHD1L + ZKSCN3 cells. (Scale bars = 100 μm). **C** Visualize statistics of tumor metastases (**p < 0.01, ***p < 0.001; Mean ± SD, = 5/group).

To further verify the finds in vivo, QGY-7703-Vec, QGY-7703-CHD1L and QGY-7703-CHD1L + ZKSCAN3 cells were established and intrasplenically inoculated into the nude mice (Fig. 6A). Histopathological examination of the mice liver was performed to examine tumor metastasis 6 weeks after tumor inoculation. As shown in the represented figures, CHD1L overexpression strongly induced metastatic tumor nodule formation in mice liver (5/5), whereas, this effect was impaired by overexpression of ZKSCAN3 (1/5) (Fig. 6B, C). No metastatic tumor nodule was observed in mice injected with QGY-7703-Vec cells.

### CHD1L-ZKSCAN3 axis promotes HCC migration potentially through autophagic degradation of Paxillin

**Fig. 7:**
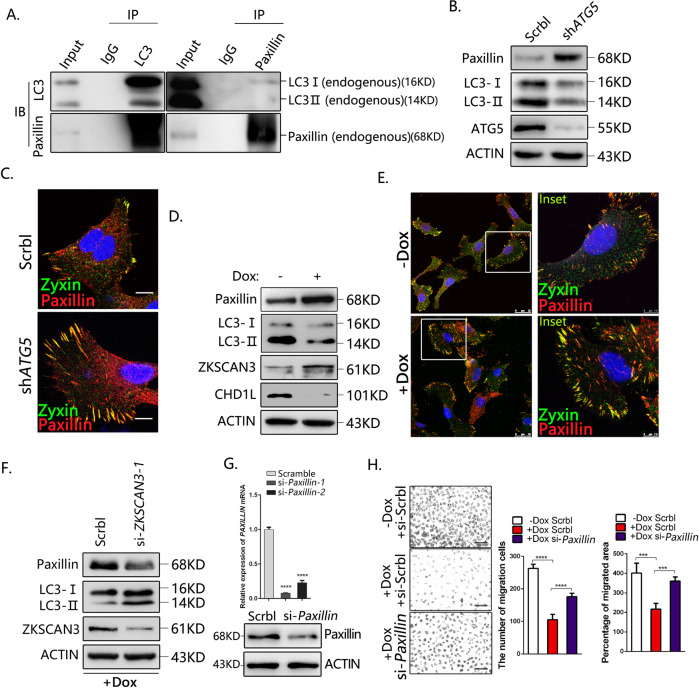
CHD1L-ZKSCAN3 axis promotes HCC migration partially through autophagic degradation of Paxillin. **A** Co-IP assays of interaction between LC3B and Paxillin in QGY-7703 cells. The precipitates were examined with anti-Paxillin and anti-LC3B antibodies, respectively. **B** WB analysis for Paxillin accumulation in QGY-7703 cells treated with Sreamble or shRNA for *ATG5*. **C** Immunofluorescence of FA proteins Paxillin and Zyxin in control and ATG5 silencing cells. Scale bars: 10 μM. **D** WB analysis for Paxillin accumulation in QGY-7703 cells treated with or without Dox. **E** Immunofluorescence of FA proteins Paxillin and Zyxin in QGY-7703 cell with or without Dox inducing. Scale bars represent as shown. **F** Paxillin accumulation defects in CHD1L-depleted cells in response to *ZKSCAN3* knockdown. Images/immunoblots are representative of three independent experiments. **G** The mRNA and protein level of Paxillin was analyzed in QGY-7703 cells treated with the most efficient siRNA fragment for *Paxillin* (hereafter referred to as si-*Paxillin*), compared with the scrambled control. **H** QGY-7703 cells with Dox treated were subjected to wound-healing assay and migration in response to *Paxillin* knockdown, compared with the scrambled control. Quantification of migration indicated group (****p < 0.0001, Student *t* test; values are shown as mean ± SD calculated from three parallel experiments; n = 5). Scale bar as indicated.

Paxillin is a FA adapter protein that plays distinct roles in the regulation of cell movement, cancer development, and metastasis [36–41]. A recent study demonstrated that Paxillin interacts with processed LC3 through a conserved LIR motif in the amino-terminal end, and is responsible for FA turnover, tumor cell motility, and metastasis [6]. Our co-immunoprecipitation (IP) results confirmed that the endogenous Paxillin binds with LC3B in QGY-7703 cells (Fig. 7A). To investigate whether Paxillin is involved in CHD1L-mediated HCC tumor cell migration, *ATG5*-specific shRNA was used to block autophagy. Both western blot (Fig. 7B) and the IF staining (Fig. 7C) demonstrated that blocking autophagy increased the accumulation of Paxillin. IF staining for FA components Paxillin and Zyxin demonstrated the significant increase of both FA size and number in autophagy-deficient cells, implying that autophagy suppression may affect the morphology of FAs through Paxillin degradation. WB analysis indicated that deletion of CHD1L significantly induced Paxillin accumulation (Fig. 7D). However, the mRNA level of Paxillin was not affected by CHD1L, indicating that the regulation of CHD1L on Paxillin was through post-transcriptional modification (Fig. S3B). IF staining for the FA proteins Paxillin and Zyxin demonstrated the increased FA number and size in CHD1L-deficient cells (Fig. 7E). These findings suggested that CHD1L-mediated autophagy-lysosome pathway may degrade Paxillin, and further affect the morphology of FAs. To determine whether CHD1L-regulated Paxillin degradation was dependent on ZKSCAN3, Paxillin level was examined in CHD1L-deficient cells treated with *ZKSCAN3*-specific siRNA. WB analysis showed that Paxillin was significantly decreased after ZKSCAN3 downregulation (Fig. 7F). These results provided evidence for that the CHD1L modulates the autophagic degradation of Paxillin through transcriptional repression of ZKSCAN3. To ascertain the involvement of Paxillin in CHD1L-induced tumor cell migration, siRNAs were used to specifically silencing *paxillin* expression (Fig. 7G). Both wound-healing and transwell assay showed that depletion of CHD1L inhibited HCC cell migration and silencing of *paxillin* rescued the decreased cell migration induced by CHD1L depletion (Fig. 7H). The experiments were further confirmed in another HCC cell line Huh7 and the similar results were obtained (Fig. S3A). On the contrary, when using 3-MA to prevent autophagic degradation or overexpressing *paxillin*, the strong migratory ability induced by CHD1L overexpression was also significantly abolished (Fig. S3B, C). All these results demonstrated that CHD1L-ZKSCAN3 axis might promote HCC migration and metastasis potentially through autophagic degradation of Paxillin.

## Discussion

Autophagy has been regarded as a potential target for tumor therapy. Although the autophagy inhibitors have been tested in clinical trial and achieved certain efficacy, the underlined molecular mechanisms still remain poorly understood. In the present study, we report that the chromatin remodeling proteinCHD1L is critical in regulating tumor cell autophagy. Our data demonstrated that overexpression of CHD1L demonstrated a higher autophagy flux which is responsible for enhanced cell migration and metastasis. Bioinformatics prediction and experimental validation indicated that CHD1L might promote autophagy through suppressing the transcription of the critical autophagy regulator ZKSCAN3. CHD1L functions as an ATP-dependent chromatin remodeling enzyme and a transcriptional regulator in gene regulation [42]. Several downstream genes such as SPOCK1, TCTP, and ARHGEF9 are transcriptionally regulated by CHD1L through directly binding to the promoters of these relevant target genes [11, 43]. Hererin, we provided new evidences that CHD1L can specifically bind to the distal promoter region of *ZKSCAN3* and negatively regulates its transcription, which further affects HCC tumor cell autophagy.

Our further study proved that autophagy is indispensable for CHD1L-induced tumor cell migration. Inhibition of autophagy attenuates cancer cell migration and invasion via decreasing the dynamic turnover of FA [36–41]. The autophagy cargo adapter Near BRCA1 delivers multiple FA proteins to the autophagosomes [44]. Paxillin, one of the most important FA protein, may interact with autophagy-related protein LC3B and degrade through autophagy. We demonstrated that the autophagic degradation of Paxillin depends on the repression of ZKSCAN3 induced by CHD1L. In addition, CHD1L increased the conversion of LC3-II, which links Paxillin protein to autophagosomes, and finally facilitated the autophagic degradation of Paxillin and the morphology variation of FAs. We acknowledge that the dynamic regulation of FA is very complicated during cancer cell migration. Overactivation or inactivation of FA are both not good for tumor cell migration. The role of FA on cell migration might also depend on the actual steps of tumor malignant progression. In the present study, we found autophagic degradation of Paxillin induced by CHD1L is able to promote HCC cell migration under pathological conditions. We propose that degradation of Paxillin might loosen the cell-cell contact, and favor its movement ability. We do not rule out the possibility that enhanced FA might also promote cell migration/tumor metastasis under certain circumstances. Further investigation of FA turnover and Paxillin degradation on tumor cell migration/metastasis in a dose and situation-dependent manner might better elucidate the detailed molecular mechanisms. In summary, we have identified a novel CHD1L/ZKSCAN3/Paxillin autophagic axis in regulating tumor cell migration and metastasis. Overexpression of CHD1L transcriptionally suppressed the expression of autophagy inhibitor ZKSCAN3, and accelerated autophagic degradation of Paxillin, which is crucial for dynamic disassembly of FA of tumor cells. Our findings provide insights into understanding the roles of cell autophagy during tumor malignant transformation. Further inhibition of CHD1L and its downstream autophagy signaling might provide novel therapeutic strategy in cancer treatment.

## Materials and methods

### Cell culture and reagents and antibodies

Human liver carcinoma Huh7 and QGY-7703 cells were grown as previously described [8]. Their background information has been described [7, 8, 10–13]. For starvation, cells were cultured in serum-free DMEM Dulbecco’s Modified Eagle Medium with no d-Glucose and Sodium Pyruvate (Gibco). The cell line has recently been authenticated by sequencing identification method. Antibody information is listed in Supplementary Table 1. *ZKSCAN3* siRNA, *Paxillin* siRNA, and control siRNA were purchased from Ribobio, and their sequences are listed in Supplementary Table 2. 3-MA (HY-19312) were purchased from MedChemExpress. The plasmid of PXN (Genebank: NM_001080855) and its corresponding Control were provided by GENECHEM.

### Lentivirus-mediated *CHD1L/ZKSCAN3* overexpression and shATG5 knockdown

The shATG5 (hU6-MCS-Ubiquitin-EGFP-IRES-puromycin, GOSL0195542) Plasmid was constructed by GENECHEM, and cells were selected in puromycin. GFP-tagged CHD1L was cloned into pLenti6/V5-TOPO^®^vector (Life technologies, Carlsbad, CA), and cells were selected with Blasticidin (Sigma-Aldrich, St. Louis, MO). *ZKSCAN3* expression construct (*ZKSCAN3* [NM_001242894.1]/FLAG was cloned into pLV[Exp] -mCherry:T2A:Puro-EF1A) was constructed by Cyagen Biosciences Inc, and cells were selected with Ampicillin. For transient knockdown assay (siRNAs are listed in Supplementary Table 2), the siRNA against *ZKSCAN3, Paxillin*, and the scrambled sequence (Qiagen, CA, USA) was transfected to the indicated cells. Virus production and cell transduction in indicated cells were performed as previously described [6].

### Construction of Dox-induced *CHD1L* knockout cell line

The packaging plasmid pMD2.G and the envelope plasmid psPAX2 were used for Cas9 lentiviral production as previously described [45]. Huh7 and QGY-7703 cells were infected with Cas9 lentivirus, and selected with puromycin. The iCas9-Huh7 and QGY-7703 cells were transfected with PLX-mCherry-U6-*CHD1L*-SgRNA lentivirus, followed by a screen for Blasticidin resistance as well as mCherry expression. Specifically, all the transfected cells were undergoing blasticidin selection at a concentration of 5 μg/mL for 10 days and the mCherry fluorescence was detected using fluorescence microscope (Olympus IX73). The successfully established inducible *CHD1L*-KO Huh7 and QGY-7703 cell line was termed as iKO cells.

### Animal experiments

The animal experiment was approved by the Ethics Committee of the Guangzhou medical University. Female BALB/c nude mice (3–4 weeks old) were randomly divided into indicated groups and intrasplenically inoculated with indicated QGY-7703 cells respectively. All procedures were performed according to institutional standard guidelines of the Committee on the Use of Live Animals in Teaching and Research of Guangzhou Medical University, Guangdong, China. Tumor metastasis was monitored for a period of 6 weeks [29]. Mice were sacrificed and the livers were paraffin-embedded and analyzed by H&E staining or for further analysis.

### Migration assays

Transwell (24 mm diameter, 8 µm pores, Costar, Corning, NY) was performed as standard protocol. Finally, five random fields from each Transwell sample were selected to examine under a light microscope. Each experimental group was repeated three times.

### Wound-healing assay

Wounds were generated using a sterile pipette tip when the cultured cells reached a density of 90% confluence. The cell culture medium was then replaced with a serum-free DMEM and wound closure was monitored over a 24- or 48-h period with a phase contrast microscope at ×200 magnification.

### Immunofluorescence

The cells were seeded on poly-lysine coated chamber slides, and subjected to starvation or treatment as indicated. The cell slides were fixed (methanol or 4% paraformaldehyde), permeablized (0.1% TritonX-100), blocked (10% Goat serum), and incubated with primary antibody in PBS overnight at 4 °C and with secondary antibody for 1 h at room temperature, counterstained with DAPI, mounted and visualized using a confocal microscope (Leica, Wetzlar, Germany).

### Transmission electron microscopy

The different groups of 7703-iKO cells were harvested, fixed with a solution containing 3% glutaraldehyde in 0.1 M cacodylate buffer, pH 7.3, for 1 h. Then, the samples were washed and treated with 0.1% Millipore-filtered cacodylate buffered tannic acid, postfixed with 1% buffered osmium tetroxide for 30 min, and stained with 1% Millipore-filtered uranyl acetate. Afterward, the samples were washed, dehydrated, embedded in 618 epoxy resin, and cut into ultrathin sections. The sections were stained with uranyl acetate and counterstained with lead citrate. The autophagic vacuoles (autophagosomes and autolysosomes) were observed using transmission electron microscope (Hitachi7000, Hitachi, Japan) at a 60-kV acceleration voltage. Count of the autophagic vacuoles from 10 cells per group was obtained for quantitation and statistics.

### Real-time PCR

Real-time PCR was performed as described previously [8]. The mRNA levels were determined and normalized against 18s mRNA. The sequences of primers are listed in Supplementary Table 3.

### Chromatin immunoprecipitation assay (ChIP assay)

The ChIP experiments were performed using the ChIP-kit from Thermo Fisher (Catalog No. 26156) following the manufacturer’s protocol as described previously [8]. Input (in each qRT–PCR reaction) was used to normalize the values. All ChIP assays were repeated twice and individual qPCR reactions were performed in triplicate with results presented as average values ± SD. Sequences of primers are listed in Supplementary Table 4.

### Co-IP and WB analysis

Co-IP was performed by following the manufacturer’s protocol (Thermo Fisher, catalog No. 26149). WB was performed as previously described [8].

### Luciferase assay

Cells were plated in 24- or 48-well plates 24 h before transfection. All plasmids were co-transfected with a control Renilla luciferase plasmid (pRL-TK). The ratio of experimental plasmid to control plasmid was 50:1. Luciferase assays were performed with the Dual-Luciferase Reporter Assay system (Promega). Cell lysates were transferred in triplicate to 96-well plates and analyzed on a Skanlt software 3.2 (Thermo Scientific) according to the manufacturer’s instructions. The firefly luminescence signal was normalized to the Renilla luminescence signal.

### MTS assay

For each group indicated as this study, 1000 cells were seeded in 96-well plates, and discard the medium in the well, 100 μL DMEM and 20 μL MTS (Promega, USA) were added to each well and incubated for 2 h at 37 °C. The optical density of the cultures was measured at wavelengths of 490 nm using a V Max kinetic microplate reader at different time periods. Each experiment was performed in triplicate.

### Colony formation

Colony formation was performed as standard protocol. After removing the staining solution, masses of ≥ 50 cells were counted as individual colonies under a bright-light microscope.

### Immunohistochemical analysis

Immunohistochemistry or H&E staining was performed as described previously [8]. The primary antibodies were used as indicated.

### Statistics

Data are represented as mean ± SD. The statistical analyses were performed using Student’s *t* test to compare two groups. The *p* values less than 0.05 were considered as significant. All statistical analyses were performed using SPSS 17.0 software.

## Supplementary information

Table S1

Table S2

Table S3

Table S4

supplementary figure legends

supplementary Figure 1

supplementary Figure 2

supplementary Figure 3

## Data Availability

All data generated or analyzed during this study are included in this published article [and its supplementary information files].
