# Supplementary material for: CHD1L augments autophagy-mediated migration of hepatocellular carcinoma through targeting ZKSCAN3
Source: Cell Death Dis. 2021 Oct 15;12(10):950. doi: 10.1038/s41419-021-04254-x (PMC8520006; doi:10.1038/s41419-021-04254-x)
Supplement: Supplementary file 2 — Table S2 [file 41419_2021_4254_MOESM2_ESM.pdf]

Table 1. Sequence information for siRNA used in described studies:

Paxillin siRNA#1	Sence 5'- GCAGCAACCUUUCUGAACUdTdT -3'
Paxillin siRNA#1	Antisence 5'- AGUUCAGAAAGGUUGCUGCdTdT -3'
ZKSCAN3 siRNA#1	Sence 5'- GGUAGCUUCUAGGCUUACUdTdT -3'
ZKSCAN3 siRNA#1	Antisence 5'- AGUAAGCCUAGAAGCUACCdTdT -3'
CHD1L siRNA#1	Sence 5'- CCUGCUGGAUAAGCUACUAdTdT -3'
CHD1L siRNA#1	Antisence 5'- UAGUAGCUUAUCCAGCAGGdTdT -3'
CHD1L siRNA#2	Sence 5'- CCAAACUGCAGCUCACCAAdTdT -3'
CHD1L siRNA#2	Antisence 5'- UUGGUGAGCUGCAGUUUGGdTdT -3'
ATG5 siRNA#1	Sense: 5'- CCAUCAAUCGGAAACUCAUTT-3'
ATG5 siRNA#1	Antisense: 5'-AUGAGUUUCCGAUUGAUGGTT-3'
