# Supplementary material for: CHD1L augments autophagy-mediated migration of hepatocellular carcinoma through targeting ZKSCAN3
Source: Cell Death Dis. 2021 Oct 15;12(10):950. doi: 10.1038/s41419-021-04254-x (PMC8520006; doi:10.1038/s41419-021-04254-x)
Supplement: Supplementary file 3 — Table S3 [file 41419_2021_4254_MOESM3_ESM.pdf]

Table 2. Sequence information for real time PCR primers used in described studies:

Paxillin	F-	GATTCATCCACCAGCAGCCTCAG
Paxillin	R-	GCACGGAGAGCCAACACTGTC
ZKSCAN3	F-	GCACGGAGAGCCAACACTGTC
ZKSCAN3	R-	AGTGTGGATTCTGTGATGTTCAAGGAG
CHD1L	F-	GGGATTCACCTACGCTCTTACC
CHD1L	R-	CCTTGTCGCCTGCATATGTTAC
18S	F-	AACCCGTTGAACCCCATT
18S	R-	CCATCCAATCGGTAGTAGCG
MAP1LC3B	F-	CTGTTGGTGAACGGACACAG
MAP1LC3B	R-	ACAATTTCATCCCGAACGTC
WIPI2	F-	GGACTCTGCCTCTCACGACT
WIPI2	R-	ACCCAGGTCGTCTGTGTAGG
SYNTAXIN5	F-	CAGACCCGTCAGAATGGAAT
SYNTAXIN5	R-	TGGCAAATGTGTTGCTAAGG
YKT6	F-	GGCCGAACTAGATGAGACCA
YKT6	R-	AGACTGTGTTCCCAGCACCT
RELA	F-	AGGCTCCTGTGCGTGTCTCC
RELA	R-	TCGTCTGTATCTGGCAGGTACTGG
DYNC2H1	F-	TCGAGCTCATGCTCTCTTCA
DYNC2H1	R-	CCAGCCCGAAGATCTGATAA
SAPK1	F-	AGATCCCGGACAAGCAGTTA
SAPK1	R-	GCTGCCCTCTTATGACTCCA
COPZ1	F-	ACAAGACCCATCGGACTGAC
COPZ1	R-	CATAGGAGCTGCCAATCACA
ARL5B	F-	GTGGGATATTGGTGGTCAGG
ARL5B	R-	AGTCGTTCCCTGTCAATGCT
AKT1S1	F-	AGAAGCAGGAGGAGGAGGAG
AKT1S1	R-	CAAACTCGTTCATGGTCACG
