# Supplementary material for: CHD1L augments autophagy-mediated migration of hepatocellular carcinoma through targeting ZKSCAN3
Source: Cell Death Dis. 2021 Oct 15;12(10):950. doi: 10.1038/s41419-021-04254-x (PMC8520006; doi:10.1038/s41419-021-04254-x)
Supplement: Supplementary file 4 — Table S4 [file 41419_2021_4254_MOESM4_ESM.pdf]

Table 3. Sequence information for ChIP-PCR primers used in described studies:

ZKSCAN3 DP1	F- TGGTGTTTAAGGCACAGTGGGA
ZKSCAN3 DP1	R- TGTCATGTGTTTACTGTCCTTCCA
ZKSCAN3 DP2	F- AGGACAGTAAACACATGACATAGCA
ZKSCAN3 DP2	R- AGGTTCAAGCTCGGGACCTTC
ZKSCAN3 DP3	F- GGTCCCGAGCTTGAACCTCA
ZKSCAN3 DP3	R- CTTGAGGGACTGCAGCCTCA
GAPDH	Comes from the ChIP kit (Thermo Fisher. Catalog No.26156)
