# Supplementary material for: CHD1L augments autophagy-mediated migration of hepatocellular carcinoma through targeting ZKSCAN3
Source: Cell Death Dis. 2021 Oct 15;12(10):950. doi: 10.1038/s41419-021-04254-x (PMC8520006; doi:10.1038/s41419-021-04254-x)
Supplement: Supplementary file 5 — supplementary figure legends [file 41419_2021_4254_MOESM5_ESM.docx]

***Supplemental figure legends***

***Figure S1.***

1. Western-blotting analysis for ATG5 in Scrbl shRNA and *ATG5* shRNA-expressing QGY-7703 clones both in indicated group.
2. QGY-7703 cells treated as indicated (with PBS control, Baf A1 (40 nM), CQ (25uM)) were plated in the upper chamber of the filters for 24 h and then, the cells migrated to the underside of the Transwell insert were counted, scale bar: 100 μM. QGY-7703 cells treated as indicated were subjected to wound-healing assay (Left). Quantification of migration in Transwell assay and migrated area in wound-healing assay (Right) (*** *p* < 0.001; ns, no significant; Student t test; n = 10).

***Figure S2.***

1. QGY-7703 cells were detected for expression of ZKSCAN3 by fluorescent quantitative RT-PCR.
2. Western-blotting analysis for LC3 conversion and P62 level changed in QGY-7703 cells in responding to ZKSCAN3 knockdown by si*ZKSCAN3*-1, as indicated to perform, compared to Scramble control.
3. Western-blotting analysis for the core member of mTOR signaling pathway in QGY-7703 cells in responding to CHD1L-deletion.
4. Western-blotting analysis for LC3B in response to ZKSCAN3 knockdown in CHD1L deletion Huh7 and QGY-7703 cells treated with Baf A1 (6 h/80 nM) under starvation (8 h).

***Figure S3.***

**A.** Representative images showing Huh7 iKO cells with Dox treated were subjected to Wound-healing assay and migration in response to *Paxillin* knockdown, compared with the scrambled control. Scale bar: 100 μM. **B, C.** Representative images showing QGY-7703 cells treated as indicated (with PBS control, 3-MA (2mM) or overexpressing Paxillin ) were plated in the upper chamber of the filters for 24 h and then, the cells migrated to the underside of the Transwell insert, scale bar: 100 μM.
